# Phosphatase Complex Pph3/Psy2 Is Involved in Regulation of Efficient Non-Homologous End-Joining Pathway in the Yeast *Saccharomyces cerevisiae*

**DOI:** 10.1371/journal.pone.0087248

**Published:** 2014-01-31

**Authors:** Katayoun Omidi, Mohsen Hooshyar, Matthew Jessulat, Bahram Samanfar, Megan Sanders, Daniel Burnside, Sylvain Pitre, Andrew Schoenrock, Jianhua Xu, Mohan Babu, Ashkan Golshani

**Affiliations:** 1 Department of Biology, Carleton University, Ottawa, Ontario, Canada; 2 Ottawa Institute of Systems Biology, Carleton University, Ottawa, Ontario, Canada; 3 Department of Biochemistry, Research and Innovation Centre, University of Regina, Regina, Saskatchewan, Canada; 4 Department of Computer Science, Carleton University, Ottawa, Ontario, Canada; 5 College of Pharmaceutical Sciences, Zhejian University, Hangzhou, Zhejiang, China; St. Georges University of London, United Kingdom

## Abstract

One of the main mechanisms for double stranded DNA break (DSB) repair is through the non-homologous end-joining (NHEJ) pathway. Using plasmid and chromosomal repair assays, we showed that deletion mutant strains for interacting proteins Pph3p and Psy2p had reduced efficiencies in NHEJ. We further observed that this activity of Pph3p and Psy2p appeared linked to cell cycle Rad53p and Chk1p checkpoint proteins. Pph3/Psy2 is a phosphatase complex, which regulates recovery from the Rad53p DNA damage checkpoint. Overexpression of Chk1p checkpoint protein in a parallel pathway to Rad53p compensated for the deletion of *PPH3* or *PSY2* in a chromosomal repair assay. Double mutant strains *Δpph3/Δchk1* and *Δpsy2/Δchk1* showed additional reductions in the efficiency of plasmid repair, compared to both single deletions which is in agreement with the activity of Pph3p and Psy2p in a parallel pathway to Chk1p. Genetic interaction analyses also supported a role for Pph3p and Psy2p in DNA damage repair, the NHEJ pathway, as well as cell cycle progression. Collectively, we report that the activity of Pph3p and Psy2p further connects NHEJ repair to cell cycle progression.

## Introduction

Among DNA lesions, double-stranded DNA breaks (DSBs) are regarded as the most severe form of DNA damage. The mechanisms for DSB repair are divided in two independent pathways, Homologous Recombination (HR), and Non-Homologous End Joining (NHEJ). HR utilizes an undamaged homologous template, preferably the sister chromatid or homologous chromosomes, to repair the broken sites of DSBs [1], [2], and is considered to be an error free repair pathway [3]. A more flexible alternative to the HR repair system is NHEJ [4], [5]. In NHEJ, the two broken strands of DNA can be ligated directly. Because NHEJ does not use a homologous template, there is a higher risk of errors in repair, which can result in mutations [6]. NHEJ is the main pathway to repair DSBs in mammals [7].

The NHEJ pathway is highly conserved from yeast to human. Yku70p and Yku80p are *S. cerevisiae* homologs of Ku70p and Ku80p, respectively, which bind to DSB ends; they form a ring which is required as a factor for protecting and stabilizing the broken ends of DNA from degradation. The MRX (Mre11p, Rad50p, Xrs2p) complex in yeast is homologous to MRN (Mre11p, Rad50p, Nbs1p) in mammalian cells. It forms a bridge between the two broken ends of DNA and brings the broken ends closer to each other preparing them for ligation. The MRX complex is recruited by Yku70/Yku80 to the site of a DNA break. It is thought that Xrs2p is one the key protein for targeting of the MRX complex to the damage site, although both the complex and all individual members of the complex can bind to DNA directly [8], [9]. Recent evidence suggests that MRN may function in multiple steps of NHEJ in mammalian cells [10]. The Dnl4/Lif1 complex is the homolog of mammalian DNA ligase XRCC4 which has ligase activities. Lif1p interacts with Xrs2p and Dnl4p, and Dnl4p performs the ligation of DNA [11], [12], [13]. Nej1p binds to the Dnl4/Lif1 complex through an interaction with Lif1p. Although its exact role is still unclear, recent investigations suggest it is recruited to the site of break, interacts with DNA and participates in the final steps of ligation [14]. Plasmid repair analyses have demonstrated that NEJ1p is required for NHEJ to function at high efficiency [14].

The efficiency of NHEJ depends on a growing number of factors. For example, different histone acetyltransferases are shown to be required for NHEJ efficiency [15], [16]. Another study reported that NHEJ is dependent on different stages of the cell cycle; NHEJ activity NHEJ is higher in G1 compared to G2/M [17]. In a recent study, methylation of histone H3 lysine 36 was shown to enhance the efficiency of NHEJ [18].

Before committing to mitosis, cells pass through different cell cycle checkpoints. Checkpoints can be activated in response to DNA damage, incomplete DNA replication and damaged replication complexes. By recognizing DNA damage and regulating cell cycle arrest, they delay cell cycle progression to provide additional opportunity for DNA repair. Defects in checkpoint function can cause genomic instability [19]. Temporal association between the cell cycle and DNA damage is thought to begin with Mec1p, a DNA damage dependent checkpoint gene [20]. Mec1p phosphorylates Rad9p [21], [22]. Phosphorylation of Rad9p further stimulates the activity of Mec1p to trigger several kinases including Rad53p and Chk1p [23], [24], [25]. The checkpoint Rad53p is a key protein in response to DNA damage. Activation of Rad53p up-regulates repair genes, down-regulates cyclins and delays cell cycle progression. It is shown that the phosphatase complex Pph3/Psy2 negatively regulates Rad53p activity by dephosphorylating it and allowing cell cycle progression to continue [26]. Recently, it was shown that the deletion of *PPH3* reduced the ability of cells to complete DSB repair via HR [27].

Here, we report that the deletion of *Pph3* and *Psy2* reduces the efficiency of NHEJ in *S. cerevisiae*. We further illustrate that this activity appears connected to cell cycle regulation.

## Materials and Methods

### Yeast Strains and Plasmid

The yeast strains are gene deletion variants of S288C *(*MAT ***a*** orfΔ::kanMX4 his3Δ1 leu2Δ0 met15Δ0 ura3Δ0 ), described in [28]. JKM139 (MATa hmrΔ::ADE1 hmlΔ::ADE1 ade1-100 leu2-3,112 lys5 trp1::hisG ura3-52 ade3::GAL-HO) strain is described in [29], [30]. Yeast mating type alpha strain Y7092 (MATa can1Δ::STE2pr-HIS3 lyp11Δ ura31Δ 0 leu21Δ0 his31Δ1 met151Δ0) was used for mating experiments [31]. For plasmid repair assay a derivation of plasmid p416 with a LacZ insert following the GAL promoter region [32] was used. Gene knockouts are produced by transformation with a PCR product containing a NAT selection gene as described in [28]. The DNA damage (DD) array was generated on the basis of GO term by arraying gene deletion mutants for 384 genes with known or potential involvement in DNA damage response, DNA replication, cell cycle progression or localization in nucleus. Overexpression plasmids which used are 2-micron plasmid as explained in [33].

### Plasmid Repair Assay

A unique XbaI restriction site was used for plasmid (p416) linearization and the repair assay was performed as in [15]. Each experiment was repeated at least five times.

### Chromosomal Repair Assay

Chromosomal double stranded breaks were induced by exposing the cells to galactose. Serial dilutions of cells (10^−3^–10^−5^) were exposed to galactose for 90 minutes to induce HO endonuclease and compared to those before exposure. Number of colonies formed before and after induction of HO endonuclease was used as a measure of survival and the efficiency of the cell to repair induced DSBs. Each experiment was repeated at least five times. For compensation experiments, gene overexpression in a single mutant background was generated by transforming the JKM139-based gene deletion strains using a corresponding plasmid carrying the target gene [33], or an empty vector as a control. To study NHEJ efficiency in different phases of the cell cycle, cells were synchronized in G1, S, and G2/M phases using drug treatment with 10 µg/ml alpha-factor, 0.2 M hydroxyurea, and 15 µg/ml nocodazole, for 2.5 hours, before exposure to galactose.

### Drug Sensitivity Spot Test

A series of single and double deletion mutants grown to mid-log were diluted (10^−2^–10^−5^) and spots of 15 µl of each dilution were placed on YPD plates containing 60 mM hydroxyurea (HU), 4 µg/ml bleomycin, or no drug as a control. Reduced colony size and numbers represented increased sensitivity.

### Genetic Interaction Analysis

Genetic interaction between target genes and DNA damage array (DDA) was examined using a miniaturized version of Synthetic Genetic Array (SGA) analysis [34]. In miniaturized SGA (mSGA) a target gene is deleted or overexpressed (plasmid-based), in an alpha mating type strain and crossed to two arrays of 384 gene deletion strains, one for target genes (DD array) and the other random (as a control) [32]. Double mutant strains were scored for fitness as in [35], [36] with some modifications. In brief, average colony size (S_ave_) was calculated by summing the size of all colonies on a plate and dividing by the total number (384). S_ave_ was subtracted from each colony to derive a relative size for individual colonies. Each experiment was repeated three times and those colonies that had a reduction of 30% or more in two of the three repeats were deemed “positive”. Synthetic sick interactions (positives) were categorized as follows: moderate (30–49% reduction), strong (50–69%), and very strong (70–99%), as in [37]. For conditional interactions, the above analysis was repeated in the presence of low (sub-inhibitory) concentrations of DNA damage-inducing drugs. Hits were confirmed by random sporulation. Synthetic dosage lethality (SDL) analysis was performed as above with the exception that overexpression plasmids were transformed into the above deletion arrays as in [37]. Gene classification on the basis of cellular process and function was performed by Yeast Features (http://software.dumontierlab.com/yeastfeatures/), Yeast Genome Database (http://www.yeastgenome.org/) and GeneMANIA (http://www.genemania.org/).

### Protein-Protein Interaction Prediction

Protein-Protein Interactions (PPIs) were predicted on the basis of co-occurring polypeptide regions as in [38]. An updated high confidence PPI database (approximately 55,000 interactions) was generated from published data (BioGRID: www.thebiogrid.org and DIP: www.dip.doe-mbi.ucla.edu). The analysis was performed at 99.95% specificity (a measure for false positive prediction) generating a sensitivity (percentage of interactions that can be identified from the total interactions that a protein makes) of 28% (in comparison to the sensitivity of 14.6% in [38] estimated by leave-one-out analysis. The local regions that mediate PPIs were predicted using PIPE-site algorithm [39].

## Results and Discussion

### Deletions of PPH3 and PSY2 Reduced the Efficiency of NHEJ in a Plasmid Based Repair Assay

To evaluate the activity of Pph3/Psy2 complex on the efficiency of NHEJ, a plasmid repair assay was utilized [40], [41]. Equal amounts of circular and linearized plasmids were transformed separately to both wild-type and deletion mutants for *PPH3* and *PSY2*. Transformed cells were plated on a selective media in a way that only cells receiving an intact plasmid or cells capable of repairing a received digested plasmid would form a colony. In this case, DNA repair is limited to NHEJ because the break site has no homologous region within the genome of *S. cerevisiae.* The number of colonies formed from linearized plasmids is related to colonies formed from intact plasmids, and this ratio reflects the proportion of successful NHEJ events that have occurred. Previously, using this assay, deletion effects for the *RSC2*, a member of the RSC, chromatin remodeling complex [40], *SIR2*, *SIR3*, *SIR4* proteins involved in telomere maintenance [41], and yeast histone acetyltransferase *RTT109* have been evaluated [15]. Deletion of *YKU70* or *YKU80* reduced NHEJ efficiency to approximately 6% and has been used as a positive control [40], [15].

**Figure 1:**
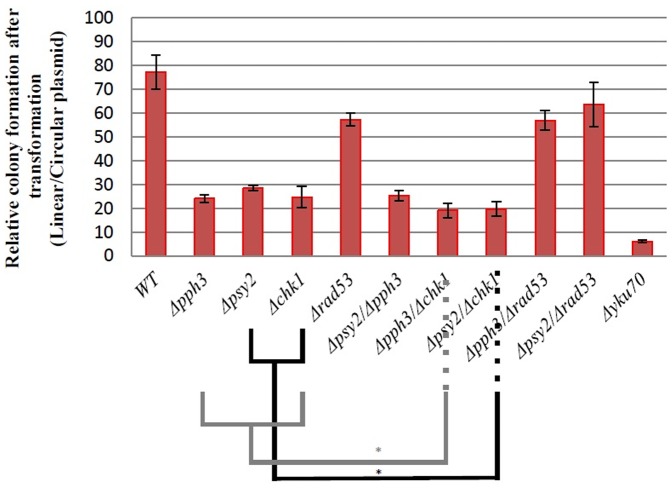
Plasmid repair efficiency for different yeast strains. Each experiment was repeated at least five times. Error bars represent standard deviation. *indicates P value of <0.05. *Δyku70* was used as a positive control.

It was observed that efficiency of NHEJ for individual deletions of *PPH3* and *PSY2* was approximately 24% and 28%, respectively (Figure 1). Deletion of both *PPH3* and *PSY2* had a NHEJ efficiency of approximately 25%. This data is in agreement with the involvement of Pph3/Psy2 phosphatase complex in efficient NHEJ of a plasmid DNA.

### The Effect of Pph3p and Psy2p on Efficient NHEJ is Relevant in a Chromosomal Context

**Figure 2:**
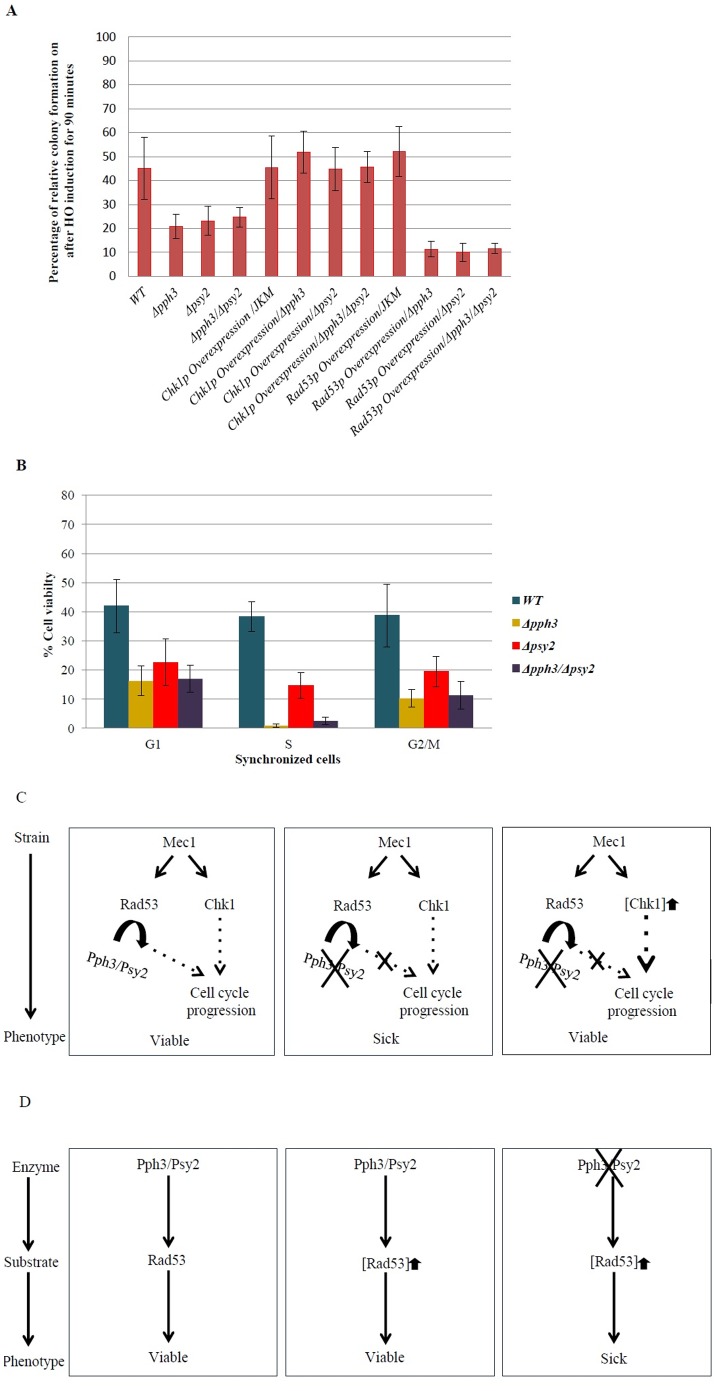
Phenotypic analysis of JKM139-based strains. (A) Fraction of the colonies that grew after HO endonuclease induction. Deletion mutant for *PPH3* or *PSY2* had reduced survival and recovered when *CHK1* was overexpressed. Overexpression of *RAD53* reduced survival when *PPH3* or *PSY2* were deleted. (B) Fraction of the colonies that grew after HO endonuclease induction when cells were synchronized in G1, S or G2/M phases. (C) Illustration of conceptual basis for the observed activity of Pph3/Psy2 complex and Chk1p in parallel pathways. (D) Illustration of conceptual basis for the observed activity Pph3/Psy2 complex (enzyme) in relationship to Rad53p (substrate). Overexpression of the substrate in the absence of the enzyme can result in a very sick phenotype. [X]↑ refers to overexpression of gene X.

We subsequently sought to confirm the involvements of Pph3p and Psy2p in efficient NHEJ in a chromosomal context, using a JKM139 strain-based chromosomal break assay [29]. In this assay, the target genes are knocked-out in a JKM139 strain background and the viability of target gene deletion mutants are evaluated after exposure to galactose. JKM139 strain carries a GAL promoter in front of an endonuclease specific to the HO site. The presence of galactose induces the production of this endonuclease and consequently results in chromosomal breakage at the HO sites. Wild-type, *Δpph3,* and *Δpsy2* cells (JKM139 background) were exposed to DSB inducing conditions for 90 minutes and allowed to form colonies (Figure 2A). Fractions of colonies formed before and after exposure to galactose were used as a measure of survival and were related to the ability of the cell to repair induced DSBs (Figure 2A). As expected, *Δpph3* and *Δpsy2* strains had a reduced ability to survive when DSBs were induced compared to wild-type, further supporting the involvement of Pph3p and Psy2p in the efficiency of NHEJ.

Cell cycle dependency for *Δpph3* and *Δpsy2* strains was investigated by synchronizing the cells in G1, S, and G2/M phases by treating the cells with alpha-factor, hydroxyurea, and nocodazole, respectively, before HO endonuclease induction. It was observed (Figure 2B) that *Δpph3* and *Δpsy2* strains had their lowest NHEJ efficiencies in S phase (1% and 14%, respectively). The significant reduction in the efficiency of NHEJ for *Δpph3* strain appears to separate the activity of *PPH3* from *SPY2* during S phase. A possible explanation is that in S phase, in addition to its *SPY2*-dependent activity, *PPH3* might also affect NHEJ efficiency through an additional pathway, which is independent of *PSY2*.

### The Pph3/Psy2 Complex Functions in Association with Components of Cell Cycle

The *PPH3/PSY2* complex is associated with a cell cycle checkpoint through dephosphorylation of the checkpoint protein Rad53p [26], [27]. Above, we showed that deletion of individual and both members of this complex reduced efficiency in NHEJ as measured by plasmid repair analysis. To determine if these results are in fact associated with checkpoint activity, the activity of other related checkpoint proteins was investigated for their effect on NHEJ using plasmid repair assay. We observed that NHEJ efficiency for deletion of *CHK1* was 25%. Deletion of *RAD53* [42], [43], which works in parallel with *CHK1*, reduced NHEJ efficiency to 57% (Figure 1).

Double mutant strains *Δpph3/Δchk1* and *Δpsy2/Δchk1* showed NHEJ efficiency of 19% and 20%, respectively. NHEJ efficiency for *Δpph3/Δrad53* and *Δpsy2/Δrad53* double mutant strains were 57% and 64%, respectively, which were similar to that for *Δrad53* (57%) single mutant suggesting that the effect of these two genes on NHEJ is likely within the same pathway as Rad53p. In this context, Rad53p appears to be upstream of Pph3p and Psy2p that the activity of these two proteins is dependent on the presence of Rad53p. A possible explanation is that deletion of *RAD53* triggers a second parallel pathway, for example Chk1p-dependent pathway, which works independent of Pph3p and Psy2p. This second parallel pathway is not triggered when *RAD53* is intact.

### Overexpression of CHK1 can Recover DNA Damage Sensitivity Phenotypes in Δpph3 and Δpsy2 Mutants in JKM139

We also used the JKM139 strain to detect phenotypic compensation in a chromosomal assay. Overexpression of genes in the DNA damage repair pathways was evaluated for its ability to compensate for a phenotype caused by deletion of *PPH3* and *PSY2*. In this way, genes that have compensating functions can be identified.

It was observed that overexpression of *CHK1* compensated for the absence of either *PPH3* or *PSY2* in a JKM139 assay (Figure 2A). Such a recovery provides a strong support for a functional association for *CHK1* with *PPH3* and *PSY2.* This is explained by the activity of Chk1p being in a parallel pathway which is compensatory to that of Pph3p and Psy2p, in response to activation of Mec1 (Figure 2C). Of interest, overexpression of *RAD53* had a compounding effect on phenotypes of *PPH3* and *PSY2* deletions (Figure 2A and 2D). Deletion strains for *PPH3* and *PSY2* grew very poorly (sick phenotype) if *RAD53* was overexpressed when DSB was induced. This observation is in accordance with the assumption that a certain equilibrium between “enzyme and substrate” can be important for cell viability [33] (Figure 2D). Rad53p (substrate) is known to be dephosphorylated by Pph3/Psy2 complex (enzyme). In this context, overexpression of the substrate in the absence of the enzyme caused a conditional sick phenotype. Overexpression of *CHK1* or *RAD53* alone did not affect the phenotype of a wild-type JKM139 strain.

### Drug Sensitivity Analysis

**Figure 3:**
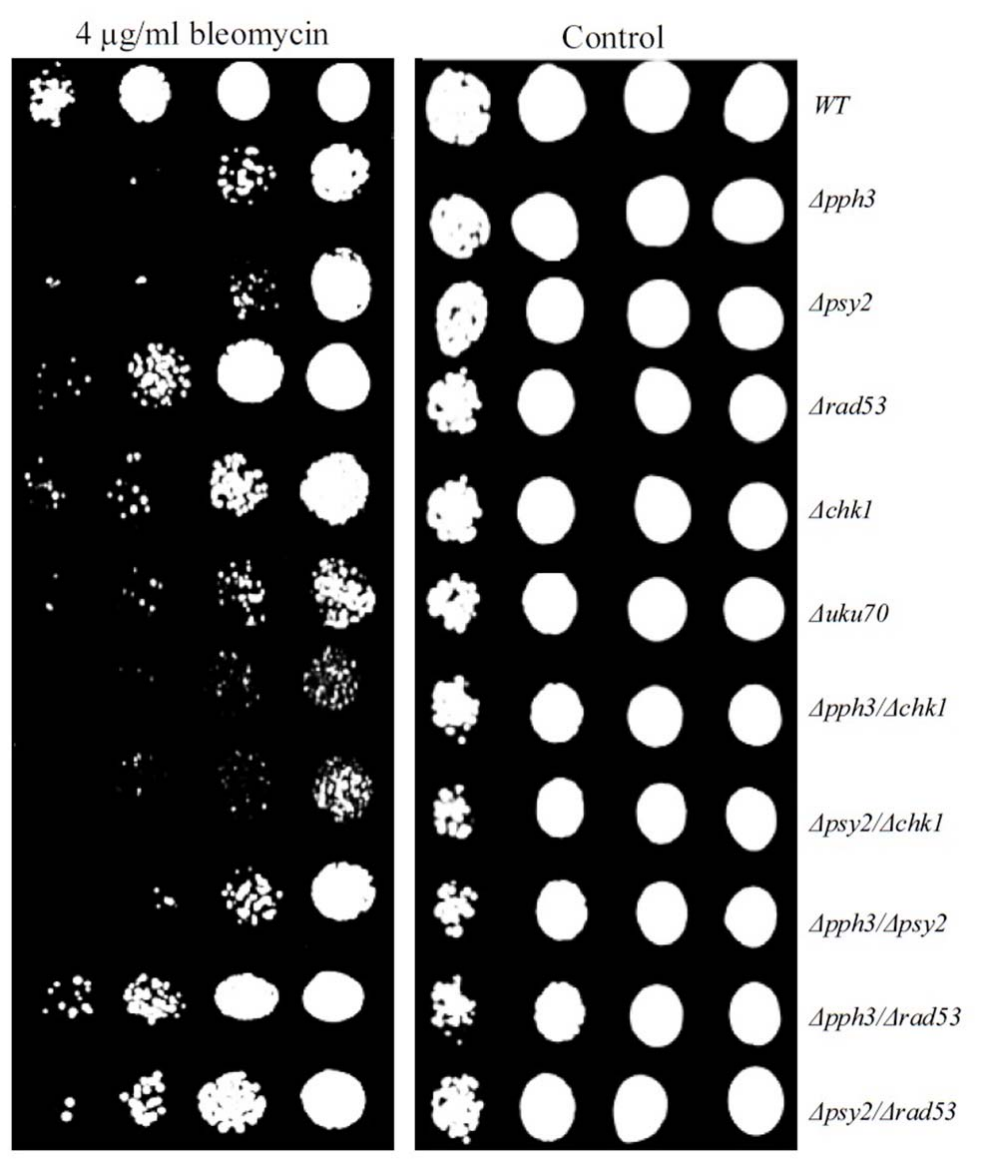
Strain sensitivity analysis to bleomycin. Single deletion mutants for *PPH3* or *PSY2* showed increased sensitivity to DSB inducing agent bleomycin. Double deletion mutant strains *Δpph3Δchk1* and *Δpsy2Δchk1* had elevated sensitivity in comparison to single deletion mutants *Δpph3* and *Δpsy2.* In contrast double deletion mutant strains *Δpph3Δrad53* and *Δpsy2Δrad53* had reduced sensitivity in comparison to single deletion mutants *Δpsy2* and *Δpph3*.

It is expected that deletion of genes involved in DNA repair pathway might change (usually elevate) the sensitivity of their deletion strains to DNA damage-inducing drugs. We used drug sensitivity to bleomycin and hydroxyurea (HU), to further study the activity of Pph3p and Psy2p. Bleomycin causes DSBs via a free-radical mechanism, and HU generates DNA replication errors that can lead to DSB [44], [45]. *Δrad53* strain showed sensitivity to HU (Figure S1) and *Δpph3, Δpsy2, Δchk1* and *Δrad53* strains all showed increased sensitivity to bleomycin (Figure 3), confirming previously reported observations [46], [26], [27]. Double mutant strains *Δpph3Δchk1* and *Δpsy2Δchk1* had elevated sensitivity in comparison with single mutants. This is in agreement with the activity of Chk1p in a parallel pathway to Pph3p and Psy2p. Double deletion mutants *Δpph3Δrad5*3 and *Δpsy2Δrad53* showed similar sensitivity to bleomycin as *Δrad53*. This can be explained by the activity of Pph3p and Psy2p which is dependent on the presence of Rad53p, as above.

### Genetic Interactions Analysis for PPH3 and PSY2

**Figure 4:**
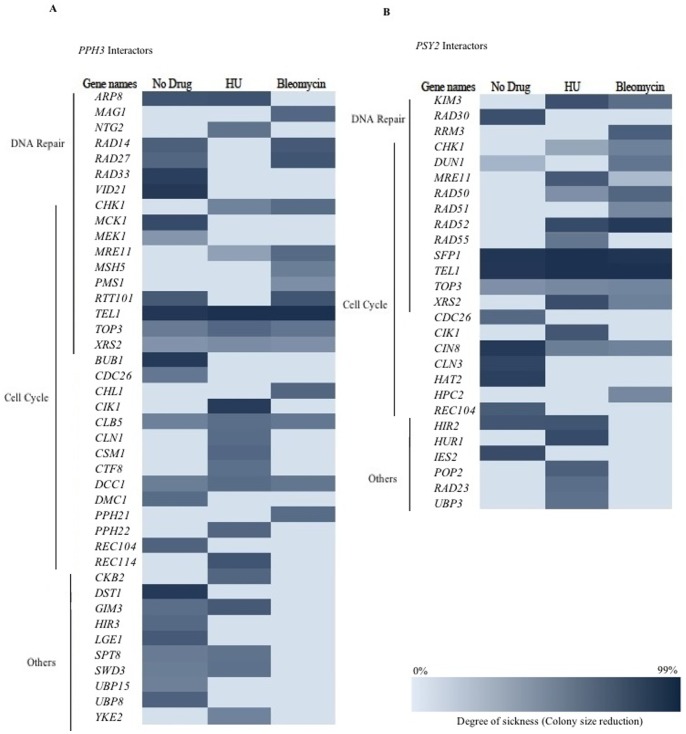
Analysis of the synthetic sick interactions for *PPH3* (A) and *PSY2* (B). Most of the interactors are involved in DNA repair and/or cell cycle progression. Conditional interactions were identified in the presence of sub-inhibitory concentrations of HU (45 mM) or bleomycin (3 µg/ml).

A genetic interaction refers to phenotypes of overexpression/deletion of two genes together that are not easily explained by the investigation of two single genes alone [47]. It reveals a higher order pathway association between genes and their functions. Since functionally related genes often genetically interact with one another [48], one way that the function(s) of a gene is studied is through the genetic interactions that it makes with other genes with known functions. In this context, genetic interactions are divided into two groups of negative and positive interactions. A more extreme phenotype for a double mutant than expected infers a negative or aggravating interaction, whereas in positive or alleviating interaction the phenotype of the double mutant is less severe. A negative genetic interaction is often observed when two genes interact through parallel pathways [49]. To further study the activity of *PPH3* and *PSY2* we examined their negative genetic interactions under standard laboratory growth condition and in the presence of sub-inhibitory concentrations of DNA damaging agents bleomycin and HU. In this way, conditional genetic interactions were investigated when DNA damage was induced. We used the method of synthetic genetic array (SGA) analysis [34] to examine sick phenotypes (negative interactions) for two mini-arrays, one for DNA damage (DD) which is a collection of 384 deletion strains for genes associated with DNA damage response, DNA replication, cell cycle progression and other interesting genes whose products are localized to nucleus, and a second array that contains 384 random deletion strains, used as a control. Using a DD array, 25 and 12 synthetic sick interactions were observed for *PPH3* and *PSY2* respectively (Figure 4) in comparison to 4 and 3 in a random array. Illustrated in Figure 4 on the basis of their cellular process, the interacting genes can be grouped into two categories of cell cycle progression or DNA repair (or both) connecting the activity of *PPH3* and *PSY2* to both cell cycle progression and DNA repair with P values of 2.65×10^−11^ and 2.95×10^−27^ for *PPH3* and 8×10^−13^ and 5.98×10^−10^ for *PSY2*, respectively, with the assumption that random array represents the global distribution of negative interactions. This is in agreement with the enrichment of negative interactions previously reported for *PPH3*, 6.78×10^−9^ and 5.73×10^−6^, and *PSY2*, 5.73×10^−6^ and 2.3×10^−4^, for response to DNA damage and cell cycle progression, respectively [50], [51]. Differences in the genetic interaction profiles for *PPH3* and *PSY2* may underscore their additional functions within the cell that are independent of each other. For example, unlike *PSY2*, *PPH3* does not form negative genetic interactions with HR genes, suggesting that a previously reported role for *PPH3* in HR [27] appears independent of *PSY2*.

Presence of sub-inhibitory concentrations of bleomycin (3 µg/ml; MIC = 7.5 µg/ml) or HU (45 mM; MIC = 150 mM) generated a number of previously unreported conditional negative interactions (Figure 4). As expected, majority of these new interactions are linked to the DNA damage response. For example, *MAG1* encodes for a 3-methyl-adenine DNA glycosylase that initiates base excision repair, *PMS1* encodes for a mismatch repair protein, and *XRS2* is a DSB repair protein, among others. Of interest, *CHK1* formed a conditional negative genetic interaction with both *PPH3* and *PSY2* in the presence of bleomycin. In the presence of HU, *CHK1* also interacted with both *PPH3* and *PSY2*. These conditional interactions are in agreement with a DNA damage dependent functional association for Pph3p and Psy2p with Chk1p.

**Figure 5:**
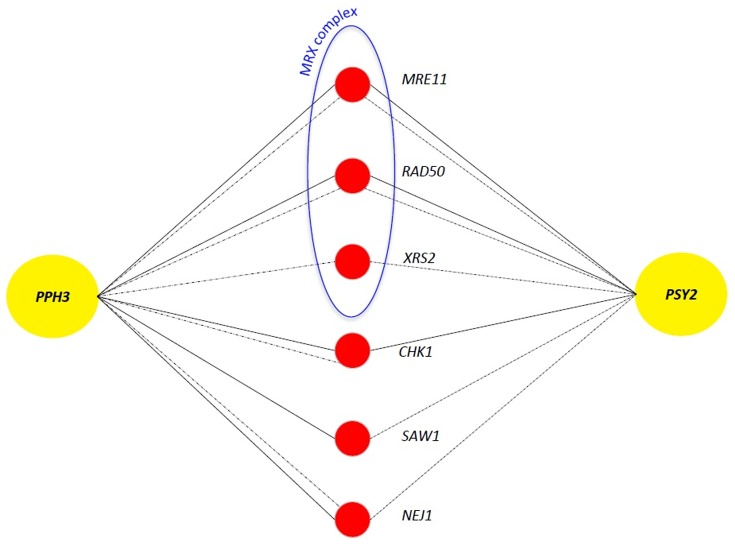
Synthetic dosage lethality (SDL) analysis. Overexpression of *PPH3* and *PSY2* formed conditional SDL interactions with members of MRX complex, in addition to *CHK1*, *SAW1* and *NEJ1.* Solid and dashed lines represent interactions found in the presence of bleomycin (3 µg/ml) and HU (45 mM), respectively.

Overexpression of certain genes may have no phenotypic consequence for a wild-type strain, however when a second gene is deleted, the same overexpression may result in an unexpected phenotype such as sickness or lethality. This type of interaction is termed Synthetic Dosage Lethality (SDL) [52], [53] and is often used to study the relationship between regulator and substrate where the overexpression of the substrate in the absence of the regulator often causes a severe phenotype [54], [55]. To study potential regulators for the Pph3/Psy2 complex, we examined the overexpression phenotypes for *PPH3* and *PSY2* on DD array in the presence and absence of sub-inhibitory concentration of DNA damage drugs bleomycin and HU as above. In the absence of DNA damage, overexpression of *PPH3* or *PSY2* did not form any SDL interactions. However, when DNA damage was induced, overexpression of either *PPH3* or *PSY2* formed SDL interactions with gene deletion strains for each of the three members of MRX complex *MRE11*, *RAD50* and *XRS2* (P value of 5.43×10^−16^) (Figure 5). This data is in agreement with a DNA damage dependent regulation of Pph3/Psy2 by MRX complex. MRX is an evolutionarily conserved complex that recognizes and binds DSBs and regulates the activity of the major DSB response kinases Mec1p and Tel1p. Since Pph3/Psy2 complex dephosphorylates activated Rad53, regulation of Pph3p and Psy2p by MRX complex may explain previous finding that the activity of Mre11p was found linked to accumulation of phosphorylated Rad53p [56], [57]. In light of our finding here, a plausible model is that MRX complex might promote the activity of Pph3p and Psy2p to dephosphorylate activated Rad53p and hence regulate cell cycle progression during DNA damage. This model merits further investigation. Of interest, *CHK1* was also found as a conditional interacting partner when either *PPH3* or *PSY2* was overexpressed. These SDL interactions further connect the activity of Chk1p with Pph3p and Psy2p during DNA damage and suggest that the Pph3/Psy2 complex might also be under the conditional regulation of Chk1p kinase. Other genes that formed conditional SDL interactions with *PPH3* and *PSY2* are *SAW1* and *NEJ1.* Saw1p facilitates the binding of Rad1/Rad10 complex to the site of DNA damage during HR [58] and Nej1p is a regulator of NHEJ [59].

### Protein-Protein Interaction Prediction

Proteins often realize their function through interactions with one another. The overall profiles of such interactions can reveal information about the function as well as the cellular process in which proteins participate. Some protein-protein interactions (PPIs) are mediated by a finite number of short interaction motifs [60], [61]. Such interactions can be studied by examining the co-occurrence of small polypeptide regions which are significantly enriched in the dataset of high confidence interacting proteins [38], [62]. One advantage of this method is that the polypeptide regions which are responsible for a physical interaction between proteins can be identified [39]. Here, we examined the possible proteome-wide interactions that Pph3p and Psy2p make on the basis of short interacting motifs. Their predicted interaction partners, along with their proposed site of interactions, are represented in Table 1. A potential interaction between amino acids 16–231 for Pph3p and 317–337 for Psy2p was identified. Previously, Pph3p was reported to interact with Psy2p [26] however the region responsible for this physical association remained unclear. Similarly, interactions between Pph3p (amino acids 298–336) and Rad53p (amino acids 266–268), as well as Psy2p (amino acids 587–608) and Rad53p (amino acids 298–336) were proposed. A number of these interactions appear to be competing for the same binding site on Pph3p and Psy2p. Such competing interactions may function as regulators of activity. For example, Rrd1p, a cell cycle regulator that activates PP2A phosphatase, competes for the same region of Pph3p (amino acids 213–286) as Rad53p. Rrd1p abundance is reported to increase in response to DNA damage [63] and can potentially outcompete the interaction of Pph3p with Rad53p, preventing the dephosphorylation of Rad53p by Pph3p in response to DNA damage. Further investigations are needed to examine the validity of this model of regulation for Pph3/Psy2 activity. The interacting partners of Pph3p and Psy2p can be grouped into two general categories of DNA damage response and cell cycle progression. These PPI profiles are in agreement with the activity of Pph3/Psy2 in regulating the cell cycle in response to DNA damage.

**Table 1:** Protein-Protein interaction prediction for Pph3p and Psy2p. Of the 24 proteins that interact with Pph3p, 6 have a role in DNA repair, 8 in both DNA repair and regulation of cell cycle, and 10 in “other” cellular processes.

Gene Names	Site of Interaction	Description
**Pph3 Interacting Partners**		
HTA1*	213–286	Core histone protein; DNA damage-dependent phosphorylation by Mec1p facilitates DNA repair
HTA2*	213–286	Core histone protein; DNA damage-dependent phosphorylation by Mec1p facilitates DNA repair
SPT4*	201–248	Regulation of transcription elongation; transcription-coupled DNA repair
SPT5*	56–136	Component of the universally conserved Spt4/5 complex; has a role in transcription-coupled DNA repair
TIP41*	28–74	Regulator of PP2A pathway; protein abundance increases in response to DNA replication stress
*TDH2*	56–136	Glyceraldehyde-3-phosphate dehydrogenase; protein abundance increases in response to DNA replication stress
HTB1*	56–253	Core histone protein required for chromatin assembly; regulates meiotic DSB formation
HTB2*	56–253	Core histone protein required for chromatin assembly; regulates meiotic DSB formation
*PSY2*	16–231	Subunit of protein phosphatase PP4 complex; regulates recovery from the DNA damage checkpoint
PSY4*	213–248	Regulatory subunit of protein phosphatase PP4; recovery from the DNA damage checkpoint
RAD53*	266–286	Protein kinase required for cell-cycle arrest in response to DNA damage
RRD1*	213–286	Peptidyl-prolyl cis/trans-isomerase involved in G1 phase progression, and DNA repair
*SRS2*	60–136	DNA helicase and DNA-dependent ATPase involved in DNA repair and checkpoint recovery
*TDH1*	56–136	Glyceraldehyde-3-phosphate dehydrogenase; protein abundance increases in response to DNA replication stress
*CCT2*	56–156	Subunit beta of the cytosolic chaperonin Cct ring complex, related to Tcp1p/required for the assembly of actin and tubulins in vivo
*CCT3*	56–253	Subunit of the cytosolic chaperonin Cct ring complex, related to Tcp1p/required for the assembly of actin and tubulins in vivo
*DIA4*	31–73	Probable mitochondrial seryl-tRNA synthetase
*PRO1*	26–46	Gamma-glutamyl kinase; catalyzes the first step in proline biosynthesis
SSD1*	215–242	Translational repressor with a role in polar growth and cell wall integrity
*STE12*	56–136	Transcription factor that is activated by a MAP kinase signaling cascade
*TAP42*	56–138	Essential protein involved in the TOR signaling pathway
*TCP1*	56–253	Alpha subunit of chaperonin-containing T-complex, which mediates protein folding in the cytosol
*TDH3*	77–97	Glyceraldehyde-3-phosphate dehydrogenase involved in glycolysis and gluconeogenesis
*YHR033W*	213–243	Protein of unknown function
**Psy2p Interacting Partners**		
HTA1*	121–152	Core histone protein; DNA damage-dependent phosphorylation by Mec1p facilitates DNA repair
HTA2*	121–152	Core histone protein; DNA damage-dependent phosphorylation by Mec1p facilitates DNA repair
*KSP1*	587–608	Serine/threonine protein kinase; protein abundance increases in response to DNA replication stress
*MCK1*	587–608	Dual-specificity serine/threonine and tyrosine protein kinase; Involved in control of chromosome segregation and in regulating entry into meiosis
SPT4*	405–430	Regulation of transcription elongation; transcription-coupled DNA repair
SPT5*	590–612	Component of the universally conserved Spt4/5 complex; has a role in transcription-coupled DNA repair
TIP41*	543–565	Regulator of PP2A pathway; protein abundance increases in response to DNA replication stress
*WSS1*	145–179	Protein of unknown function; has a suggested role in the DNA damage response
HTB1*	405–428	Core histone protein required for chromatin assembly; regulates meiotic DSB formation
HTB2*	405–428	Core histone protein required for chromatin assembly; regulates meiotic DSB formation
*PHO85*	587–608	Cyclin-dependent kinase; involved in regulating the cellular response to nutrient levels and environmental conditions and progression through the cell cycle
*PPH3*	317–337	Catalytic subunit of protein phosphatase PP4 complex; regulates recovery from the DNA damage checkpoint
PSY4*	815–836	Regulatory subunit of protein phosphatase PP4; recovery from the DNA damage checkpoint
RAD53*	587–608	Protein kinase required for cell-cycle arrest in response to DNA damage
RRD1*	422–442	Peptidyl-prolyl cis/trans-isomerase involved in G1 phase progression, and DNA repair
*AAD6*	590–614	Putative aryl-alcohol dehydrogenase involved in oxidative stress response
*ARG82*	590–612	Inositol polyphosphate multikinase; diphosphoinositol polyphosphate synthase activity
*BEM2*	143–170	Rho GTPase activating protein (RhoGAP) involved in the control of cytoskeleton organization and cellular morphogenesis
*GDS1*	568–588	Protein of unknown function
*HEF3*	154–177	Translational elongation factor EF-3; stimulates EF-1 alpha-dependent binding of aminoacyl-tRNA by the ribosome
*PGA2*	143–179	Essential protein required for maturation of Gas1p and Pho8p; involved in protein trafficking
*PGC1*	467–487	Phosphatidylglycerolphosphate synthase; catalyzes the synthesis of phosphatidylglycerolphosphate from CDP-diacylglycerol and sn-glycerol 3-phosphate
*RPC25*	12–36	RNA polymerase III subunit C25; required for transcription initiation
SSD1*	155–179	Translational repressor with a role in polar growth and cell wall integrity
*STD1*	154–182	Protein involved in control of glucose-regulated gene expression
*YEF3*	154–177	Gamma subunit of translational elongation factor eEF1B; stimulates the binding of aminoacyl-tRNA (AA-tRNA) to ribosomes

Of the 26 that interacted with Psy2p, 8 have a role in DNA repair, 7 in both DNA repair and regulation of cell cycle, and 11 in “other” cellular processes. * are proteins that interact with both Pph3p and Psy2p.

## Concluding Remarks

In this study, we show that interacting proteins Pph3p and Psy2p affect the efficiency of NHEJ in the unicellular budding yeast *S. cerevisiae*. Deletion of either *PPH3* or *PSY2* genes reduced NHEJ efficiency both in the context of chromosomal and plasmid DSB repair. Pph3p and Psy2p form a phosphatase complex, which dephosphorylates Rad53 checkpoint kinase [26]. Our analyses using a plasmid repair assay suggested a functional connection between the activity of Pph3p and Psy2p on NHEJ through checkpoint protein Rad53. Similarly, phenotypic suppression analysis revealed that overexpression of Chk1p, another checkpoint kinase that works in parallel to Rad53p, compensated for the absence of either *PPH3* or*/*and *PSY2* genes in a chromosomal based repair assay. Double deletion mutant strains for either *PPH3* or *PSY2* with CHK1 showed additional reduction in the efficiency of plasmid repair through NHEJ than single mutant. Our genetic interaction analyses revealed synthetic sick phenotypes for both *PPH3* and *PSY2* with DNA damage response genes that function in regulation and upstream to DNA damage repair pathway, in addition to genes involved in cell cycle progression. This observation is in clear agreement with the activity of Pph3/Psy2 in cell cycle progression and further indicates that their effect on NHEJ is not at the mechanistic but rather at the regulatory level. This is consistent with previously reported activity of Pph3p in HR pathway [27]. In support of a role for Pph3/Psy2 in regulation of DNA damage response via cell cycle, the PPI analysis reported here suggested that both Pph3p and Psy2p interact with both DNA damage response and cell cycle progression proteins.

Dephosphorylation of Rad53p by Pph3/Psy2 releases cell cycle arrest. Pph3p also dephosphorylates γH2AX which regulates DNA damage checkpoint proteins activity. This regulation is through chromatin modification [46]. A recent study by Kim et al. reported a role for Pphp3 in DSB repair through HR [27]. Here, we show that Pph3p and Psy2p also affect the efficiency of NHEJ. We also present genetic evidence for conditional cross-talk and functional associations between Pph3p and Psy2p with checkpoint kinases Rad53p and Chk1p. These associations can be triggered by bleomycin, HU and HO endonuclease.

## Supporting Information

Figure S1 **Strain sensitivity analysis to 60 mM hydroxyurea (HU).**
(PDF)
