# Supplementary figures and images for: Phosphatase Complex Pph3/Psy2 Is Involved in Regulation of Efficient Non-Homologous End-Joining Pathway in the Yeast Saccharomyces cerevisiae
Source: PLoS One. 2014 Jan 31;9(1):e87248. doi: 10.1371/journal.pone.0087248 (PMC3909046; doi:10.1371/journal.pone.0087248)

Supplementary Figure 1

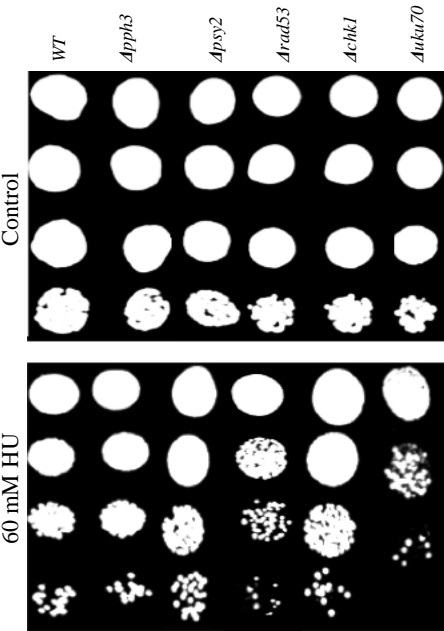

Supplement: Figure S1 — Strain sensitivity analysis to 60 mM hydroxyurea (HU). (PDF) [file pone.0087248.s001.pdf]
